# Lack of association between genetic polymorphisms within *DUSP12 - ATF6 *locus and glucose metabolism related traits in a Chinese population

**DOI:** 10.1186/1471-2350-12-3

**Published:** 2011-01-06

**Authors:** Cheng Hu, Rong Zhang, Congrong Wang, Xiaojing Ma, Jie Wang, Yuqian Bao, Kunsan Xiang, Weiping Jia

**Affiliations:** 1 Shanghai Diabetes Institute, Shanghai Key Laboratory of Diabetes Mellitus, Shanghai Clinical Center for Diabetes, Department of Endocrinology and Metabolism, Shanghai Jiao Tong University Affiliated Sixth People's Hospital, Shanghai, PR China

## Abstract

**Background:**

Genome-wide linkage studies in multiple ethnic populations found chromosome 1q21-q25 was the strongest and most replicable linkage signal in the human chromosome. Studies in Pima Indian, Caucasians and African Americans identified several SNPs in *DUSP12 *and *ATF6*, located in chromosome 1q21-q23, were associated with type 2 diabetes.

**Methods:**

We selected 19 single nucleotide polymorphisms (SNPs) that could tag 98% of the SNPs with minor allele frequencies over 0.1 within *DUSP12-ATF6 *region. These SNPs were genotyped in a total of 3,700 Chinese Han subjects comprising 1,892 type 2 diabetes patients and 1,808 controls with normal glucose regulation.

**Results:**

None of the SNPs and haplotypes showed significant association to type 2 diabetes in our samples. No association between the SNPs and quantitative traits was observed either.

**Conclusions:**

Our data suggests common SNPs within *DUSP12*-*ATF6 *locus may not play a major role in glucose metabolism in the Chinese.

## Background

Type 2 diabetes is a complex disease caused by both genetic and environmental factors. Although recent genome-wide association studies have identified several novel, possibly causative genes, the contribution of them to disease risk is still very limited [1]. Thus the genetic architecture of type 2 diabetes remained largely unknown. Previous genome-wide linkage studies in multiple ethnic populations, including Caucasians, Chinese and Pima Indian, showed that chromosome 1q21-q25 was the strongest and most replicable linkage signal in the human chromosome [2-9]. Although genome-wide association studies show no strong association signal in this region, whether variants harbored in this region conferred modest effect to the disease are worthy to be analyzed.

Dual specificity phosphatase 12 (*DUSP12*) and activating transcription factor 6 (*ATF6*) were two neighbored genes locating on the chromosome 1q21-q23. DUSP12 is a glucokinase - associated protein identified from rat hepatic cDNA library through yeast two-hybrid, using glucokinase as bait. It may participate in glycolysis in the liver and pancreatic beta-cell through dephosphorylation of glucokinase in the cytoplasm [10]. *ATF6 *is a key sensor of endoplasmic reticulum stress. It activates unfolded protein response through regulating a group of genes encoding molecular chaperones and folding enzymes [11]. Previous studies identified several single nucleotide polymorphisms (SNPs) in this region associated with type 2 diabetes in different populations. Among them, rs2070150 (P145A) was firstly identified to be associated with type 2 diabetes in Pima Indian, while rs4579731, rs3820449 and rs10918215 were reported later in studies focusing on Caucasians and African Americans [12-15]. However, International Type 2 Diabetes 1q Consortium failed to detect any association signal on *DUSP12 *and *ATF6 *in a fine mapping study in multiethnic samples [16]. Even though, only 285 East Asian origin samples were included in the previous studies and no one analyzed the association between SNPs from this region and type 2 diabetes in large Asian samples. Therefore, we performed the present study, aiming to test if variants from *DUSP12 *and *ATF6 *played a role in the genetic susceptibility of type 2 diabetes in the Chinese.

## Methods

### Participants

In this case-control study, we recruited 3,700 unrelated Chinese Han subjects, including 1,892 case and 1,808 controls. All the subjects were eastern Chinese Han ancestry, residing in Shanghai and nearby region. In the present study, all case subjects were type 2 diabetes patients selected from Shanghai Diabetes Institute inpatient database. Control subjects were community-based populations enrolled from the Shanghai Diabetes Studies [17]. The inclusion and exclusion criteria for the cases and controls were described previously [17]. Briefly, all cases were type 2 diabetes patients defined according to 1999 WHO criteria (fasting plasma glucose ≥7.0 mmol/l and/or 2-h plasma glucose ≥11.1 mmol/l) and were treated with oral hypoglycemic agents and/or insulin. The control subjects were normal glucose tolerance defined based on fasting plasma glucose <6.1 mmol/l and 2-h plasma glucose <7.8 mmol/l. This study was approved by the institutional review board of Shanghai Jiao Tong University Affiliated Sixth People's Hospital. Written informed consent was obtained from each participant.

### Clinical measurement

All subjects underwent detailed clinical investigations, as described previously [17]. Briefly, anthropometric parameters such as height, weight, waist and hip circumference (for the control subjects only) were measured. For the control subjects, blood samples were obtained at 0 and 120 min during the oral glucose tolerance tests (OGTTs) to measure plasma glucose and serum insulin levels. Lipid profiles such as total cholesterol and triglyceride were also obtained. Insulin resistance and pancreatic β-cell function were assessed by homeostasis model assessment (HOMA) [18]. HOMA-IR = fasting insulin × fasting plasma glucose ÷ 22.5, HOMA-B = 20 × fasting insulin ÷ (fasting plasma glucose ÷ 3.5).

### SNPs selection, genotyping and quality control

We selected 19 SNPs that spanning 197 kb of *DUPS12 *and *ATF6 *region, from 10 kb 5' upstream the *DUSP12 *to 2 kb 3' downstream the *ATF6*. These SNPs could tag 98% of the SNPs with MAF over 0.1 derived from HapMap Phase III Chinese Han database under the threshold of r^2 ^≥0.7. Among them, 7 SNPs located in the coding region. The SNPs previously reported were either directly genotyped or in linkage disequilibrium (LD) with genotyped SNPs. All the SNPs were genotyped using Sequenom's MassARRAY iPLEX system (MassARRAY Compact Analyzer, Sequenom, San Diego, CA, USA). The key quality control requirements were: 1) sample call rates ≥75%; 2) SNP call rate ≥85%; 3) less than two discrepant genotypes of 100 duplicate samples; and 4) Hardy-Weinberg equilibrium test ≥0.05 in controls and cases respectively. After the quality control procedures of the genotypes, 71 individuals were excluded. And one SNP (rs3767635) failed Hardy-Weinberg equilibrium test. The average call rate for the remaining 18 SNPs was 97.5%, and the average concordance rate based on 100 duplicate comparisons for each SNP was 99.4%. Detailed information of the call rates and concordance rates for the SNPs was shown in the Additional file 1 Table S1.

### Statistical analyses

Observed genotypes were tested for fit to the expectation of Hardy-Weinberg equilibrium using χ^2 ^test. Pairwise LD was estimated from the combined data of cases and controls calulating |D'| and r^2 ^using Haploview (version 4.1) http://www.broadinstitute.org/haploview/haploview[19]. Haplotype block structure was determined using confidence interval algorithm [20] and haplotype frequencies were estimated by Expectation-Maximization algorithm [21] using Haploview (v 4.1). Allele, genotype and haplotype frequencies for cases and controls were compared using χ^2 ^test or Fisher's exact test. Odds ratios (ORs) with 95% confidence intervals (CIs) were presented. The genotype - disease association analyses were performed under the additive model adjusting age, gender and BMI as confounding factors by logistic regression. Quantitative traits with skewed distribution were natural logarithmically transformed to approximate univariate normality. Quantitative traits were analyzed under an additive genetic model by linear regression adjusted for age, sex, and BMI. All statistical analyses were performed by SAS (version 8.0; SAS Institute Inc., Cary, NC, USA) unless specified otherwise. A two-tailed *P *value <0.05 was considered significant. The allele frequencies in HapMap populations and statistic power of the SNPs were shown in the Additional file 2 Table S2.

## Results

**Figure 1:**
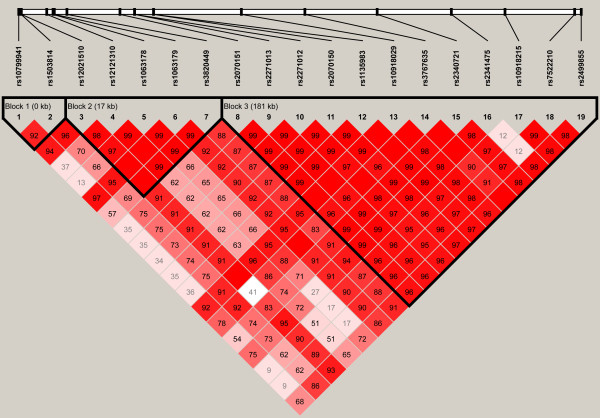
**Linkage disequilibrium plots for SNPs genotyped in *DUSP12 - ATF6 *locus in the Chinese samples**. Shades of pink indicate the strength of pairwise LD based on |D'|. Number shown are |D'| of each SNP pair.

A total of 18 SNPs were successfully genotyped in 3,629 individuals in the present study. The LD pattern of these SNPs was shown in Figure 1. Three haplotype blocks were constructed in this region.

The single SNP association analysis showed that no SNP was significantly associated with type 2 diabetes in our samples. The minimum *P *value was 0.0954 for rs10799941. Logistic regression analysis adjusting age, gender and BMI as confounding factors also suggested no association between SNPs and type 2 diabetes (Table 1). For the haplotype analysis, we compared the haplotype distributions between cases and controls and observed that no haplotype was nominally associated with type 2 diabetes (Table 2).

**Table 1:** Allele frequencies and association to type 2 diabetes for SNPs in the DUSP12-ATF6 locus

Gene	SNP	Chromosome position	Major/minor allele	Risk allele	Risk allele frequency		OR (95%CI)	*P*_allele_	*P*_genotype_
								
					Cases	Controls			
*DUSP12*	rs10799941	159974818	T,G	T	0.540	0.520	1.0821(0.9863-1.1873)	0.0954	0.1122
*DUSP12*	rs1503814	159975743	C,T	T	0.329	0.316	1.0613(0.9616-1.1714)	0.2373	0.3788
*DUSP12*	rs12021510	159975835	A,G	A	0.930	0.929	1.0141(0.8466-1.2147)	0.8794	0.9472
*DUSP12*	rs12121310	159985839	A,C	C	0.379	0.367	1.0546(0.9572-1.1620)	0.2823	0.5793
*DUSP12*	rs1063178	159988331	C,T	T	0.460	0.458	1.0074(0.9177-1.1060)	0.8766	0.7165
*DUSP12*	rs1063179	159988828	C,T	C	0.772	0.762	1.0594(0.9498-1.1817)	0.3002	0.3300
*DUSP12*	rs3820449	159993796	C,T	C	0.694	0.694	1.0002(0.9049-1.1056)	0.9964	0.6854
*ATF6*	rs2070151	160014680	C,T	T	0.311	0.300	1.0512(0.9511-1.1619)	0.3282	0.2568
*ATF6*	rs2271013	160020426	A,G	G	0.310	0.300	1.0506(0.9505-1.1612)	0.3338	0.2515
*ATF6*	rs2271012	160020465	C,T	T	0.317	0.302	1.0706(0.9666-1.1858)	0.1908	0.1373
*ATF6*	rs2070150	160027900	G,C	C	0.312	0.301	1.0568(0.9559-1.1685)	0.2805	0.2015
*ATF6*	rs1135983	160027936	C,T	T	0.309	0.297	1.0563(0.9553-1.1680)	0.2856	0.2305
*ATF6*	rs10918029	160062520	G,A	G	0.780	0.774	1.0360(0.9272-1.1575)	0.5326	0.6230
*ATF6*	rs2340721	160116009	A,C	C	0.349	0.344	1.0197(0.9247-1.1244)	0.6959	0.9399
*ATF6*	rs2341475	160145232	G,A	G	0.658	0.646	1.0531(0.9497-1.1677)	0.3268	0.4704
*ATF6*	rs10918215	160166355	A,G	G	0.432	0.427	1.0223(0.9304-1.1234)	0.6460	0.4317
*ATF6*	rs7522210	160193803	C,G	G	0.433	0.427	1.0258(0.9341-1.1264)	0.5940	0.3425
*ATF6*	rs2499855	160196385	A,G	A	0.897	0.890	1.0753(0.9203-1.2563)	0.3606	0.5117

**Table 2:** Association analyses of haplotypes in DUSP12-ATF6 locus with type 2 diabetes

Haplotype	Haplotype frequencies		*P *value
		
	Cases	Controls	
Block 1 (rs10799941-rs1503814)			

GC	0.449	0.467	0.1199
TT	0.318	0.304	0.1919
TC	0.222	0.217	0.5799
GT	0.011	0.013	0.5919

Block 2 (rs12021510-rs12121310-rs1063178-rs1063179-rs3820449)			

ACTCT	0.307	0.306	0.8928
AACCC	0.242	0.236	0.5579
AACTC	0.228	0.237	0.3592
AATCC	0.080	0.089	0.1618
GACCC	0.070	0.069	0.8911
ACTCC	0.074	0.064	0.0897

Block 3 (rs2070151-rs10918029-rs7522210-rs2499855)			

CGCA	0.342	0.341	0.9407
TGGA	0.304	0.296	0.4348
CACA	0.218	0.226	0.3917
CGGG	0.100	0.107	0.3166
CGGA	0.021	0.017	0.3030

We then analyzed the association between SNPs and quantitative traits related to glucose metabolism in the individuals with normal glucose regulation. No SNP was associated with plasma glucose and serum insulin levels at fasting status as well as 2-h after glucose stimulation. No significant association was detected between insulin sensitivity and beta cell function either (Table 3).

**Table 3:** Association between SNPs from DUSP12-ATF6 and clinical features related to glucose metabolism in the normal glucose regulation subjects

SNP	Fasting glucose			2 h glucose			Fasting insulin			HOMA-IR			HOMA-B		
	
	Beta	SE	*P*	Beta	SE	*P*	Beta	SE	*P*	Beta	SE	*P*	Beta	SE	*P*
rs10799941	0.0065	0.0164	0.6933	0.0325	0.0387	0.4024	-0.0087	0.0226	0.7019	-0.0076	0.0237	0.7492	-0.0164	0.0249	0.5116
rs1503814	0.0098	0.0179	0.5833	0.0247	0.0425	0.5613	0.0023	0.0251	0.9273	0.0065	0.0262	0.8036	-0.0135	0.0277	0.6264
rs12021510	0.0073	0.0322	0.8203	-0.0795	0.0760	0.2953	0.0039	0.0452	0.9319	0.0054	0.0473	0.9086	-0.0021	0.0499	0.9670
rs12121310	0.0288	0.0173	0.0963	0.0740	0.0411	0.0719	-0.0066	0.0241	0.7858	0.0010	0.0253	0.9678	-0.0400	0.0267	0.1346
rs1063178	0.0298	0.0165	0.0722	0.0356	0.0392	0.3639	0.0083	0.0230	0.7192	0.0162	0.0240	0.5008	-0.0259	0.0254	0.3068
rs1063179	-0.0178	0.0192	0.3541	0.0082	0.0454	0.8561	-0.0241	0.0262	0.3590	-0.0258	0.0274	0.3483	-0.0090	0.0288	0.7552
rs3820449	0.0216	0.0178	0.2256	0.0739	0.0421	0.0791	-0.0179	0.0247	0.4687	-0.0113	0.0259	0.6627	-0.0489	0.0273	0.0737
rs2070151	-0.0187	0.0184	0.3094	-0.0112	0.0435	0.7970	0.0023	0.0254	0.9295	-0.0061	0.0266	0.8182	0.0328	0.0280	0.2426
rs2271013	-0.0203	0.0184	0.2704	-0.0126	0.0435	0.7728	0.0014	0.0252	0.9559	-0.0073	0.0264	0.7822	0.0330	0.0278	0.2367
rs2271012	-0.0196	0.0186	0.2934	-0.0193	0.0440	0.6615	0.0086	0.0257	0.7376	0.0004	0.0269	0.9883	0.0376	0.0285	0.1866
rs2070150	-0.0177	0.0185	0.3397	-0.0154	0.0438	0.7249	0.0062	0.0257	0.8106	-0.0020	0.0269	0.9405	0.0349	0.0283	0.2178
rs1135983	-0.0168	0.0185	0.3633	-0.0192	0.0438	0.6609	0.0128	0.0256	0.6180	0.0046	0.0268	0.8642	0.0427	0.0283	0.1314
rs10918029	0.0075	0.0197	0.7027	0.0282	0.0467	0.5451	-0.0102	0.0271	0.7066	-0.0071	0.0284	0.8029	-0.0177	0.0300	0.5561
rs2340721	-0.0023	0.0174	0.8958	0.0488	0.0410	0.2342	-0.0074	0.0239	0.7581	-0.0064	0.0250	0.7988	-0.0125	0.0264	0.6346
rs2341475	0.0202	0.0187	0.2810	-0.0207	0.0445	0.6419	0.0021	0.0257	0.9350	0.0093	0.0269	0.7299	-0.0226	0.0289	0.4332
rs10918215	0.0022	0.0167	0.8941	-0.0441	0.0396	0.2647	0.0120	0.0232	0.6054	0.0093	0.0243	0.7006	0.0193	0.0256	0.4501
rs7522210	0.0001	0.0166	0.9945	-0.0425	0.0394	0.2809	0.0095	0.0230	0.6810	0.0061	0.0241	0.8001	0.0213	0.0253	0.3991
rs2499855	0.0153	0.0266	0.5654	-0.0842	0.0630	0.1814	0.0172	0.0367	0.6391	0.0199	0.0384	0.6045	0.0023	0.0407	0.9546

## Discussion

Genome-wide linkage studies in various populations suggested the existence of multiple susceptibility gene(s) for type 2 diabetes at chromosome 1q21-q24 [2-9]. Several specific genes in this region, such as *LMNA*, *NOS1AP *and *ATF6*, were identified that they might confer risk for diabetes in some populations [12-16,22-24]. Among these genes, *ATF6 *is a strong candidate by its biological function in endoplasmic reticulum stress and unfolded protein response, which linked insulin demand with beta cell failure and diabetes. ATF6 is also the binding target of WFS1, a known type 2 diabetes susceptible gene, and mediates its effect on endoplasmic reticulum stress [25]. However, although we performed the association study by analyzing 18 SNPs in 3700 Chinese Han, we failed to find any evidence of association between SNPs from this locus and traits related to glucose metabolism in our samples. One possible explanation might be that the statistical power of our samples was not enough to detect the effects of this locus in the Chinese population. Although we had over 80% power to detect the association at the 0.05 level based on the previously reported ORs in non-Asian populations (1.2 ~ 1.3) and allele frequencies of reported SNPs in our Chinese samples, we could not exclude the possibility the reported effect size was overestimated due to the "winner's curse" effect or novel associated SNPs with lower minor allele frequencies in the Chinese existed, in this case our samples may not have sufficient power. Secondly, the relatively loose criteria for tagging SNP selection, which is the limitation of the current study, missed information for a group of SNPs in this region. As we used r^2 ^over 0.7 and minor allele frequency over 0.1 as SNPs selection criterion, we failed to capture 15(6.8%) SNPs if the stringent criterion r^2 ^over 0.8 and minor allele frequency over 0.05 was adopted. Thirdly, the LD pattern and allele frequencies differed between Chinese Hans and previously studied populations, which suggested population differences in the genetic architecture between Chinese and other ethnic populations, may also partly explain the lack of association between this locus and previously reported phenotypes. Finally we cannot exclude the possibility that rare variants within this region participated in the pathogenesis of diabetes as we only focused on the common ones.

## Conclusion

Our data suggests common variants within *DUSP12 *and *ATF6 *genes may not play a major role in glucose metabolism in the Chinese. However, due to the limitation of the current study, the effects of SNPs from this locus on type 2 diabetes need to be tested in further studies with larger East Asian origin samples and higher marker density.

## List of abbreviations

ATF6: activating transcription factor 6; CI: confidence interval; DUSP12: dual specificity phosphatase 12; HOMA: homeostasis model assessment; LD: linkage disequilibrium; OGTT: oral glucose tolerance test; OR: odds ratio; SNP: single nucleotide polymorphism;

## Competing interests

The authors declare that they have no competing interests.

## Authors' contributions

CH designed the study, participated in genotyping, performed statistical analysis and drafted the manuscript. RZ prepared the DNA samples and participated in genotyping. CW participated in genotyping. XM participated in sample collection and clinical studies. JW participated in the clinical study and revised the manuscript. YB participated in clinical study and contributed to discussion. KX contributed to discussion. WJ supervised the study and revised the manuscript.

## Pre-publication history

The pre-publication history for this paper can be accessed here:

http://www.biomedcentral.com/1471-2350/12/3/prepub

## Supplementary Material

Additional file 1 **Call rates and concordance rates of SNPs genotyped**. This file contains detailed information of quality control analysis of the SNPs, including call rates and concordance rates. Click here for file

Additional file 2 **Allele frequencies and statistic power of the SNPs**. This file contains the allele frequencies of all SNPs in the HapMap populations and our samples. The statistic power of the SNPs in our samples was also shown in this file. Click here for file

## References

[B1] Manolio TA Collins FS Cox NJ Goldstein DB Hindorff LA Hunter DJ McCarthy MI Ramos EM Cardon LR Chakravarti A Cho JH Guttmacher AE Kong A Kruglyak L Mardis E Rotimi CN Slatkin M Valle D Whittemore AS Boehnke M Clark AG Eichler EE Gibson G Haines JL Mackay TF McCarroll SA Visscher PM Finding the missing heritability of complex diseases Nature 2009 461 7265 747 753 10.1038/nature08494 19812666 PMC2831613

[B2] Hanson RL Ehm MG Pettitt DJ Prochazka M Thompson DB Timberlake D Foroud T Kobes S Baier L Burns DK Almasy L Blangero J Garvey WT Bennett PH Knowler WC An autosomal genomic scan for loci linked to type II diabetes mellitus and body-mass index in Pima Indians Am J Hum Genet 1998 63 4 1130 1138 10.1086/302061 9758619 PMC1377493

[B3] Elbein SC Hoffman MD Teng K Leppert MF Hasstedt SJ A genome-wide search for type 2 diabetes susceptibility genes in Utah Caucasians Diabetes 1999 48 5 1175 1182 10.2337/diabetes.48.5.1175 10331426

[B4] Vionnet N Hani EH Dupont S Gallina S Francke S Dotte S De Matos F Durand E Lepretre F Lecoeur C Gallina P Zekiri L Dina C Froguel P Genomewide search for type 2 diabetes-susceptibility genes in French whites: evidence for a novel susceptibility locus for early-onset diabetes on chromosome 3q27-qter and independent replication of a type 2-diabetes locus on chromosome 1q21-q24 Am J Hum Genet 2000 67 6 1470 1480 10.1086/316887 11067779 PMC1287924

[B5] Wiltshire S Hattersley AT Hitman GA Walker M Levy JC Sampson M O'Rahilly S Frayling TM Bell JI Lathrop GM Bennett A Dhillon R Fletcher C Groves CJ Jones E Prestwich P Simecek N Rao PV Wishart M Bottazzo GF Foxon R Howell S Smedley D Cardon LR Menzel S McCarthy MI A genomewide scan for loci predisposing to type 2 diabetes in a U.K. population (the Diabetes UK Warren 2 Repository): analysis of 573 pedigrees provides independent replication of a susceptibility locus on chromosome 1q Am J Hum Genet 2001 69 3 553 569 10.1086/323249 11484155 PMC1235485

[B6] Hsueh WC St Jean PL Mitchell BD Pollin TI Knowler WC Ehm MG Bell CJ Sakul H Wagner MJ Burns DK Shuldiner AR Genome-wide and fine-mapping linkage studies of type 2 diabetes and glucose traits in the Old Order Amish: evidence for a new diabetes locus on chromosome 14q11 and confirmation of a locus on chromosome 1q21-q24 Diabetes 2003 52 2 550 557 10.2337/diabetes.52.2.550 12540634

[B7] Langefeld CD Wagenknecht LE Rotter JI Williams AH Hokanson JE Saad MF Bowden DW Haffner S Norris JM Rich SS Mitchell BD Linkage of the metabolic syndrome to 1q23-q31 in Hispanic families: the Insulin Resistance Atherosclerosis Study Family Study Diabetes 2004 53 4 1170 1174 10.2337/diabetes.53.4.1170 15047638

[B8] Ng MC So WY Cox NJ Lam VK Cockram CS Critchley JA Bell GI Chan JC Genome-wide scan for type 2 diabetes loci in Hong Kong Chinese and confirmation of a susceptibility locus on chromosome 1q21-q25 Diabetes 2004 53 6 1609 1613 10.2337/diabetes.53.6.1609 15161769

[B9] Xiang K Wang Y Zheng T Jia W Li J Chen L Shen K Wu S Lin X Zhang G Wang C Wang S Lu H Fang Q Shi Y Zhang R Xu J Weng Q Genome-wide search for type 2 diabetes/impaired glucose homeostasis susceptibility genes in the Chinese: significant linkage to chromosome 6q21-q23 and chromosome 1q21-q24 Diabetes 2004 53 1 228 234 10.2337/diabetes.53.1.228 14693720

[B10] Munoz-Alonso MJ Guillemain G Kassis N Girard J Burnol AF Leturque A A novel cytosolic dual specificity phosphatase, interacting with glucokinase, increases glucose phosphorylation rate J Biol Chem 2000 275 42 32406 32412 10.1074/jbc.M000841200 10913113

[B11] Zhang K Kaufman RJ Signaling the unfolded protein response from the endoplasmic reticulum J Biol Chem 2004 279 25 25935 25938 10.1074/jbc.R400008200 15070890

[B12] Das SK Chu WS Hale TC Wang X Craig RL Wang H Shuldiner AR Froguel P Deloukas P McCarthy MI Zeggini E Hasstedt SJ Elbein SC Polymorphisms in the glucokinase-associated, dual-specificity phosphatase 12 (DUSP12) gene under chromosome 1q21 linkage peak are associated with type 2 diabetes Diabetes 2006 55 9 2631 2639 10.2337/db05-1369 16936214

[B13] Thameem F Farook VS Bogardus C Prochazka M Association of amino acid variants in the activating transcription factor 6 gene (ATF6) on 1q21-q23 with type 2 diabetes in Pima Indians Diabetes 2006 55 3 839 842 10.2337/diabetes.55.03.06.db05-1002 16505252

[B14] Chu WS Das SK Wang H Chan JC Deloukas P Froguel P Baier LJ Jia W McCarthy MI Ng MC Damcott C Shuldiner AR Zeggini E Elbein SC Activating transcription factor 6 (ATF6) sequence polymorphisms in type 2 diabetes and pre-diabetic traits Diabetes 2007 56 3 856 862 10.2337/db06-1305 17327457 PMC2672156

[B15] Meex SJ van Greevenbroek MM Ayoubi TA Vlietinck R van Vliet-Ostaptchouk JV Hofker MH Vermeulen VM Schalkwijk CG Feskens EJ Boer JM Stehouwer CD van der Kallen CJ de Bruin TW Activating transcription factor 6 polymorphisms and haplotypes are associated with impaired glucose homeostasis and type 2 diabetes in Dutch Caucasians J Clin Endocrinol Metab 2007 92 7 2720 2725 10.1210/jc.2006-2280 17440018

[B16] Prokopenko I Zeggini E Hanson RL Mitchell BD Rayner NW Akan P Baier L Das SK Elliott KS Fu M Frayling TM Groves CJ Gwilliam R Scott LJ Voight BF Hattersley AT Hu C Morris AD Ng M Palmer CN Tello-Ruiz M Vaxillaire M Wang CR Stein L Chan J Jia W Froguel P Elbein SC Deloukas P Bogardus C Linkage disequilibrium mapping of the replicated type 2 diabetes linkage signal on chromosome 1q Diabetes 2009 58 7 1704 1709 10.2337/db09-0081 19389826 PMC2699860

[B17] Hu C Zhang R Wang C Ma X Wang C Fang Q Bao Y Xiang K Jia W A genetic variant of *G6PC2 *is associated with type 2 diabetes and fasting plasma glucose level in the Chinese population Diabetologia 2009 52 3 451 456 10.1007/s00125-008-1241-3 19082990

[B18] Matthews DR Hosker JP Rudenski AS Naylor BA Treacher DF Turner RC Homeostasis model assessment: insulin resistance and beta-cell function from fasting plasma glucose and insulin concentrations in man Diabetologia 1985 28 7 412 419 10.1007/BF00280883 3899825

[B19] Barrett JC Fry B Maller J Daly MJ Haploview: analysis and visualization of LD and haplotype maps Bioinformatics 2005 21 2 263 265 10.1093/bioinformatics/bth457 15297300

[B20] Gabriel SB Schaffner SF Nguyen H Moore JM Roy J Blumenstiel B Higgins J DeFelice M Lochner A Faggart M Liu-Cordero SN Rotimi C Adeyemo A Cooper R Ward R Lander ES Daly MJ Altshuler D The structure of haplotype blocks in the human genome Science 2002 296 5576 2225 2229 10.1126/science.1069424 12029063

[B21] Qin ZS Niu T Liu JS Partition-ligation-expectation-maximization algorithm for haplotype inference with single-nucleotide polymorphisms Am J Hum Genet 2002 71 5 1242 1247 10.1086/344207 12452179 PMC385113

[B22] Owen KR Groves CJ Hanson RL Knowler WC Shuldiner AR Elbein SC Mitchell BD Froguel P Ng MC Chan JC Jia W Deloukas P Hitman GA Walker M Frayling TM Hattersley AT Zeggini E McCarthy MI Common variation in the *LMNA *gene (encoding lamin A/C) and type 2 diabetes: association analyses in 9,518 subjects Diabetes 2007 56 3 879 883 10.2337/db06-0930 17327460 PMC2672988

[B23] Wegner L Andersen G Sparso T Grarup N Glumer C Borch-Johnsen K Jorgensen T Hansen T Pedersen O Common variation in *LMNA *increases susceptibility to type 2 diabetes and associates with elevated fasting glycemia and estimates of body fat and height in the general population: studies of 7,495 Danish whites Diabetes 2007 56 3 694 698 10.2337/db06-0927 17327437

[B24] Hu C Wang C Zhang R Ng MC Bao Y So WY Ma RC Ma X Chan JC Xiang K Jia W Association of genetic variants of *NOS1AP *with type 2 diabetes in a Chinese population Diabetologia 2010 53 2 290 298 10.1007/s00125-009-1594-2 19937226

[B25] Fonseca SG Ishigaki S Oslowski CM Lu S Lipson KL Ghosh R Hayashi E Ishihara H Oka Y Permutt MA Urano F Wolfram syndrome 1 gene negatively regulates ER stress signaling in rodent and human cells J Clin Invest 2010 120 3 744 755 10.1172/JCI39678 20160352 PMC2827948

