# Supplementary material for: Lack of association between genetic polymorphisms within DUSP12 - ATF6 locus and glucose metabolism related traits in a Chinese population
Source: BMC Med Genet. 2011 Jan 6;12:3. doi: 10.1186/1471-2350-12-3 (PMC3022799; doi:10.1186/1471-2350-12-3)
Supplement: Additional file 1 — Call rates and concordance rates of SNPs genotyped. This file contains detailed information of quality control analysis of the SNPs, including call rates and concordance rates. [file 1471-2350-12-3-S1.DOC]

Table S1 Call rates and concordance rates of SNPs genotyped

| SNP | Call rate | Discrepant genotype count | Concordant genotype count | Concordance rate |
| --- | --- | --- | --- | --- |
| rs10799941 | 97.8% | 0 | 97 | 100.0% |
| rs1503814 | 99.4% | 1 | 94 | 98.9% |
| rs12021510 | 99.1% | 1 | 94 | 98.9% |
| rs12121310 | 94.9% | 0 | 100 | 100.0% |
| rs1063178 | 97.3% | 1 | 91 | 98.9% |
| rs1063179 | 99.1% | 0 | 94 | 100.0% |
| rs3820449 | 98.4% | 0 | 100 | 100.0% |
| rs2070151 | 99.6% | 1 | 94 | 98.9% |
| rs2271013 | 98.7% | 0 | 100 | 100.0% |
| rs2271012 | 94.1% | 1 | 97 | 99.0% |
| rs2070150 | 97.6% | 1 | 98 | 99.0% |
| rs1135983 | 99.1% | 1 | 94 | 98.9% |
| rs10918029 | 98.7% | 1 | 93 | 98.9% |
| rs3767635 | 87.0% | 0 | 93 | 100.0% |
| rs2340721 | 97.5% | 1 | 99 | 99.0% |
| rs2341475 | 97.6% | 1 | 93 | 98.9% |
| rs10918215 | 97.0% | 0 | 97 | 100.0% |
| rs7522210 | 98.4% | 0 | 99 | 100.0% |
| rs2499855 | 91.6% | 0 | 97 | 100.0% |
