# Supplementary material for: Lack of association between genetic polymorphisms within DUSP12 - ATF6 locus and glucose metabolism related traits in a Chinese population
Source: BMC Med Genet. 2011 Jan 6;12:3. doi: 10.1186/1471-2350-12-3 (PMC3022799; doi:10.1186/1471-2350-12-3)
Supplement: Additional file 2 — Allele frequencies and statistic power of the SNPs. This file contains the allele frequencies of all SNPs in the HapMap populations and our samples. The statistic power of the SNPs in our samples was also shown in this file. [file 1471-2350-12-3-S2.DOC]

Table S2 Allele frequencies and statistic power of the SNPs.

|  |  |  | Frequencies of allele 1 in HapMap and our samples | | | | |  | Power of our samples | | |
| --- | --- | --- | --- | --- | --- | --- | --- | --- | --- | --- | --- |
| SNP | Allele 1 | Allele 2 | CEU | CHB | JPT | YRI | Shanghai |  | OR=1.2 | OR=1.3 |  |
| rs10799941 | G | T | 0.073 | 0.417 | 0.279 | 0.289 | 0.583 |  | 0.97 | 1 |  |
| rs1503814 | C | T | 0.225 | 0.722 | 0.591 | 0.375 | 0.722 |  | 0.93 | 1 |  |
| rs12021510 | A | G | 0.941 | 0.922 | 0.844 | 1 | 0.930 |  | 0.60 | 0.85 |  |
| rs12121310 | A | C | 0.740 | 0.611 | 0.581 | 0.570 | 0.627 |  | 0.97 | 1 |  |
| rs1063178 | C | T | 0.733 | 0.556 | 0.534 | 0.567 | 0.551 |  | 0.97 | 1 |  |
| rs1063179 | C | T | 0.933 | 0.795 | 0.911 | 0.575 | 0.767 |  | 0.89 | 1 |  |
| rs3820449 | C | T | 0.817 | 0.667 | 0.682 | 1 | 0.694 |  | 0.96 | 1 |  |
| rs2070151 | C | T | 0.965 | 0.663 | 0.605 | 0.857 | 0.694 |  | 0.96 | 1 |  |
| rs2271013 | A | G | 0.950 | 0.678 | 0.602 | 0.958 | 0.695 |  | 0.95 | 1 |  |
| rs2271012 | C | T | 0.950 | 0.678 | 0.589 | 0.842 | 0.690 |  | 0.95 | 1 |  |
| rs2070150 | C | G | 0.050 | 0.322 | 0.411 | 0.042 | 0.307 |  | 0.95 | 1 |  |
| rs1135983 | C | T | 0.957 | 0.670 | 0.593 | 0.845 | 0.697 |  | 0.96 | 1 |  |
| rs10918029 | A | G | 0.083 | 0.200 | 0.078 | 0.350 | 0.223 |  | 0.89 | 1 |  |
| rs2340721 | A | C | 0.300 | 0.644 | 0.568 | 0.975 | 0.653 |  | 0.96 | 1 |  |
| rs2341475 | A | G | 0.179 | 0.298 | 0.140 | 0.763 | 0.358 |  | 0.95 | 1 |  |
| rs10918215 | A | G | 0.783 | 0.578 | 0.456 | 0.317 | 0.570 |  | 0.97 | 1 |  |
| rs7522210 | C | G | 0.783 | 0.591 | 0.466 | 0.408 | 0.570 |  | 0.97 | 1 |  |
| rs2499855 | A | G | 0.892 | 0.889 | 0.920 | 0.983 | 0.894 |  | 0.72 | 1 |  |
